# The bed smells like oil: About a case with diagnosis of epilepsy

**DOI:** 10.1192/j.eurpsy.2021.1976

**Published:** 2021-08-13

**Authors:** M. Suárez-Gómez, P. Rodrigues, A. Suárez Gómez

**Affiliations:** 1 Psychiatry, Unidade Local de Saúde do Baixo Alentejo, Beja, Portugal; 2 Community Health, Unidade de Cuidados Continuados, Borba, Portugal

**Keywords:** old age psychiatry, Epilepsy, olfactory hallucinations, neuropsychiatry

## Abstract

**Introduction:**

Olfactory hallucinations have been described since the 19th century as a particular, often unpleasant smell at the beginning or during the spell. The olfactory cortex are involved in temporal lobe epilepsy.

**Objectives:**

The aim was analyze the relationship between the olfactory hallucinations and the previus diagnosis of epilepsy.

**Methods:**

In this study, we present a clinical case and review the current literature showing the relationship between smell and epilepsy.

**Results:**

A 69-years-old woman, with a medical history of epilepsy, went to the emergency department describing a recent episode of seizure, self-limited in time, after a sensation of an unpleasant smell in bed. A medical history of osteoarthritis, cholecystectomy and essential tremor is described. No unknown drug allergies. The neurological examination shows dysarthric speech, tremor in the right upper limb, isochoric and reactive pupils, preserved sensitivity and strength, and a positive Romber’s sign. The physical examination, blood test and vital signs were normal. The head CT scan showed signs of ischemic leukoencephalopathy, without acute ischemic or hemorrhagic lesions. The patient was medicated with 1000 mg of valproate daily, which was suspended a month ago due to an alteration in liver transaminases. Treatment with diazepam 10 mg daily was prescribed and referred for consultation. The sense of smell changes over time for anormal aging process, affecting abilitiesto detect, identify and discriminate odors.Several neurodegenerative diseases presentcertain alterations that help us determine yourorigin and progression (Vaughan and Jackson, 2014).

**Conclusions:**

Olfactory auras occurs before a seizure of the temporal lobe. Repeated stimuli in limbic regions can produce changes in the piriform cortex, with increased excitability and in epileptic discharges.

**Disclosure:**

No significant relationships.

